# Interpretations of and management actions following ECGs in programmatic cardiovascular care in primary care: A retrospective dossier study

**DOI:** 10.1007/s12471-020-01376-3

**Published:** 2020-02-19

**Authors:** N. Van den Nieuwenhof, R. T. A. Willemsen, K. T. S. Konings, H. E. J. H. Stoffers

**Affiliations:** Care and Public Health Research Institute (CAPHRI), Department of Family Medicine, Maastricht University, Maastricht, The Netherlands

**Keywords:** Electrocardiography, General practice, Quality of health care, Clinical competence, Preventive health services, Retrospective studies

## Abstract

**Background:**

The usefulness of routine electrocardiograms (ECGs) in cardiovascular risk management (CVRM) and diabetes care is doubted.

**Objectives:**

To assess the performance of general practitioners (GPs) in embedding ECGs in CVRM and diabetes care.

**Methods:**

We collected 852 ECGs recorded by 20 GPs (12 practices) in the context of CVRM and diabetes care. Of all abnormal (*n* = 265) and a sample of the normal (*n* = 35) ECGs, data on the indications, interpretations and management actions were extracted from the corresponding medical records. An expert panel consisting of one cardiologist and one expert GP reviewed these 300 ECG cases.

**Results:**

GPs found new abnormalities in 13.0% of all 852 ECGs (12.0% in routinely recorded ECGs versus 24.3% in ECGs performed for a specific indication). Management actions followed more often after ECGs performed for specific indications (17.6%) than after routine ECGs (6.0%). The expert panel agreed with the GPs’ interpretations in 67% of the 300 assessed cases. Most often misinterpreted relevant ECG abnormalities were previous myocardial infarction, R‑wave abnormalities and typical/atypical ST-segment and T‑wave (ST-T) abnormalities. Agreement on patient management between GP and expert panel was 74%. Disagreement in most cases concerned additional diagnostic testing.

**Conclusions:**

In the context of programmatic CVRM and diabetes care by GPs, the yield of newly found ECG abnormalities is modest. It is higher for ECGs recorded for a specific reason. Educating GPs seems necessary in this field since they perform less well in interpreting and managing CVRM ECGs than in ECGs performed in symptomatic patients.

**Electronic supplementary material:**

The online version of this article (10.1007/s12471-020-01376-3) contains supplementary material, which is available to authorized users.

## What’s new?

- In programmatic cardiovascular risk management (CVRM) and diabetes care, the usefulness of electrocardiograms (ECGs) seems highest in consultations in which complaints of new onset were the reason to perform the ECG.
- In the context of CVRM and diabetes care, general practitioners (GPs) perform moderately well in interpreting ECGs and managing patients following the ECG. From a previous study we know that GPs perform better in symptomatic patients.
- Education targeted at GPs’ misinterpretations observed in this study—for example R‑wave abnormalities, previous myocardial infarction, atypical ST‑T abnormalities and conduction disorders—may further improve ECG interpretation in primary care.

## Introduction

In primary care, every fourth electrocardiogram (ECG) has been reported to be performed for screening purposes in cardiovascular risk management (CVRM) [1]. Yet, the value of the ECG in primary care has been a recurrent topic of debate, above all in the context of CVRM [1–11]. The numbers needed to screen (NNS) for major outcomes have been reported to be 260 to prevent one death and 70 to find one case of coronary heart disease [2, 5, 12]. Poorly justified indications and misinterpretations, may lead to inappropriate patient management [13, 14]. Guidelines acknowledge the limited benefit of an ECG in cardiovascular risk assessment except for suspected atrial fibrillation or therapy-resistant hypertension; yet, clear guidelines on the use of ECGs in primary care settings are not available [1, 15–22].

To learn more about the use of ECGs in primary care, we conducted a series of four studies (www.nhg.org/onderzoeken/het-ecg-de-nederlandse-huisartspraktijk-0). The first study focussed on the competence of GPs in requesting and interpreting ECGs, using a case-vignette design, whereas the second study addressed the real-life performance of GPs in electrocardiography in symptomatic patients [23, 24]. In the present study, we aimed at describing the use of ECGs and the GPs’ performance in programmatic CVRM and diabetes care. In addition, we studied the use of ECGs during out-of-office hours (to be published).

## Methods

### Design and setting

A final-year medical student (N.N.) performed this retrospective dossier study during a compulsory science elective of eighteen weeks. By email, we recruited GPs who regularly perform and interpret ECGs themselves. We selected and analysed ECGs performed in the context of programmatic CVRM and diabetes care. An expert panel reviewed the interpretations and management actions of all abnormal and a random sample of normal ECGs.

### Data collection

Between September and October 2016, using email, we invited 301 GPs to participate in the study. We sent a reminder email to 173 non-responders and we approached 68 GPs by telephone. Eventually, 12 practices representing 20 GPs agreed to participate. In their practices, we included ECGs performed between 1 August 2015 and 1 August 2016 in the context of programmatic CVRM and diabetes care. Routine screening ECGs and ECGs conducted because of specific findings during the consultation were both included. All participating GPs completed a short questionnaire about personal ECG skills and usage.

Each case record consisted of a hardcopy of a 12-lead ECG, complemented with anonymised corresponding data extracted from the medical record: patient characteristics, relevant previous ECGs, indication, the GP’s current ECG interpretation, and subsequent management actions.

### Expert panel

The expert panel evaluated 300 ECGs: all ECGs that had been assessed as abnormal by the GPs (*n* = 265) and a random sample of normal ECGs (*n* = 35/587). All cases were independently assessed by one GP expert and one cardiologist. In case of a difference of opinion between both experts, a second expert GP was consulted and determined the final judgment.

Initially, without being aware of the interpretation of the study GP, the panel members provided a description of each ECG assigned to them and classified it as normal, borderline or pathologic, using a standardised ECG diagnoses list issued by the American College of Cardiology/American Heart Association (ACC/AHA) (Supplementary Table 1) [25]. Next, the panel member assessed the GP’s ECG interpretation and the subsequent clinical action and indicated to what extent (s)he agreed with the GP’s result.

### Outcome and statistics

Using Statistical Package for the Social Sciences (SPSS) version 21, we conducted descriptive analyses. Differences between groups were analysed using Pearson’s chi-square test, and *p* < 0.05 was considered statistically significant.

### Ethical considerations

We included only ECGs that had been performed more than three months before the date of inclusion. In case of severe ECG abnormalities with suggested consequences for future management, notification of the GP would take place. All patients had waived their right to give ‘notice of objection’ against the use of anonymous data for research objectives when they were given the opportunity to do so. The Medical Ethics Review Committee of Maastricht University Medical Centre waived formal review because the Medical Research Involving Human Subjects Act (WMO) does not apply to this study.

## Results

### Characteristics of the general practices and the included ECGs

**Fig. 1:**
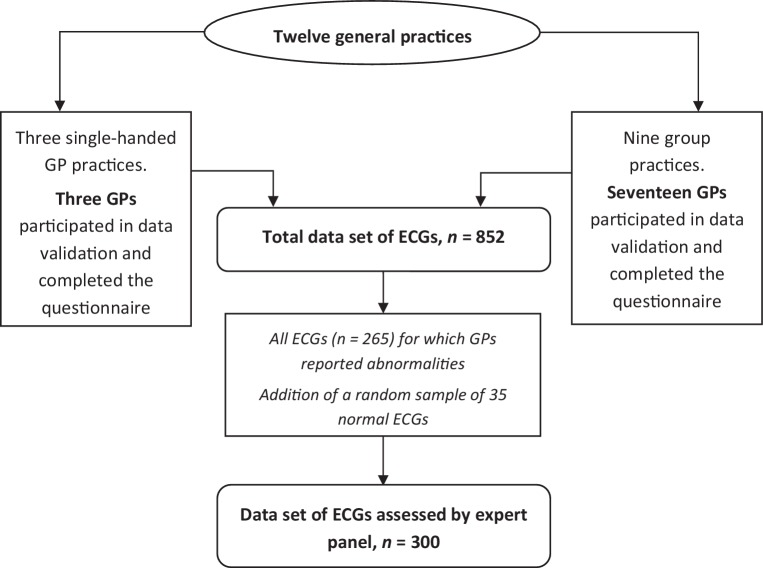
Descriptive diagram of participating general practices and included ECGs. *ECG* electrocardiogram, *GP* general practitioner

Twelve practices, representing 20 GPs who interpret ECGs themselves, participated (Fig. 1). The mean number of years of experience as a GP was 17. On average, the GPs reported interpreting 14 ECGs per month. In the context of programmatic CVRM and diabetes care, all GPs recorded ECGs ‘on indication’, 11 GPs (55%) routinely recorded ECGs when a patient entered the programme, and six GPs (30%) recorded ECGs periodically (Supplementary Table 2).

We included 852 ECG cases (mean age 66.4 years, 54% male), performed in the context of either programmatic CVRM (655 cases, 77%) or DM care (197 cases, 23%). In 22.4% of the cases, the medical history included at least one cardiovascular disease (full details: Supplementary Table 3).

### The GPs’ indications, interpretations and management actions

**Fig. 2:**
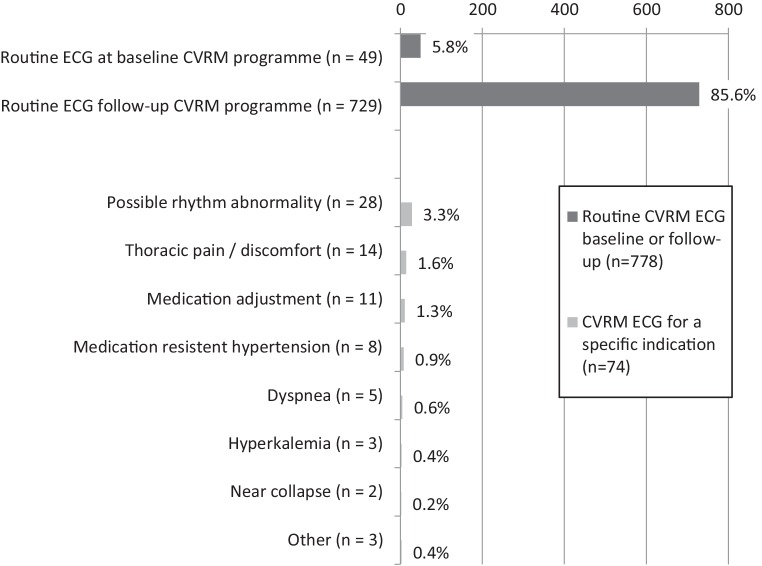
Reasons for making an ECG in CVRM (categorised in main categories: routine baseline, routine follow-up or specific indication). Absolute numbers and percentage of all ECGs (*n* = 852). Other (*n* = 3) included patient’s request, cardiologist’s request, other finding upon physical examination (each occurring once). *CVRM* cardiovascular risk management, *ECG* electrocardiogram, *GP* general practitioner

In 74/852 cases (8.7%), ECGs were performed for a specific reason; most commonly suspicion of a rhythm abnormality (*n* = 28), thoracic pain or discomfort (*n* = 14) and medication initiation or alteration (*n* = 11) (Fig. 2). The remaining ECGs were recorded routinely at entrance (*n* = 49; 5.8%) or in the course (*n* = 729; 85.6%) of the CVRM or diabetes care programme.

The GPs described abnormalities in 144/852 ECGs (16.9%) and new abnormalities in 111/852 ECGs (13.0%). Frequently reported abnormalities were left axis deviation (*n* = 75; 8.8%), suspected previous myocardial infarction (*n* = 45; 5.3%), relevant intraventricular conduction abnormalities (*n* = 42; 4.9%), atrial fibrillation or flutter (AF(l)) (*n* = 37; 4.3%, AF alone *n* = 36; 4.2%: 12 cases concerned newly discovered AF, 6 among ECGs for specific indications, 6 in routine ECGs), and atypical ST-segment and T‑wave (ST-T) abnormalities (*n* = 31; 3.6%) (Tab. 1). New abnormalities were found in 18/74 ECGs performed for a specific indication (24.3%, 95% confidence interval [CI] 16.0–35.2%), versus 93/778 ECGs performed routinely (12.0%, 95% CI 9.9–14.4%, *p* = 0.0093, Tab. 2).

**Table 1:** Occurrence of specific ECG interpretations by general practitioners

Main categories	Subcategories	Number of ECG diagnoses	
Abnormal sinus node rhythms		14	
	Sinus tachycardia (>100 beats/min)		6
	Sinus bradycardia (<50 beats/min)		8
Sinus node arrhythmias		9	
	Sinus node arrhythmia		9
	Sick sinus syndrome		0
Other supraventricular rhythms		37	
	Atrial fibrillation		36
	Atrial flutter		1
Escape rhythms & premature complexes		46	
	Premature atrial complexes (PACs)		12
	Ectopic atrial rhythm		–
	Premature ventricular complexes		34
Atrial ventricular conduction abnormalities		34	
	1st degree AV block		33
	2nd degree AV block type 2		1
Relevant intraventricular conduction abnormalities		42	
	Left bundle branch block (LBBB)		14
	Right bundle branch block (RBBB)		28
Less relevant intraventricular conduction abnormalities		53	
	Incomplete right bundle branch block (iRBBB)		27
	Incomplete left bundle branch block (iLBBB)		1
	Left anterior fascicular block (LAFB)		8
	Intraventricular conduction delay		17
Axis deviations		79	
	Left axis (+90–+180 degrees)		75
	Right axis (−30–−90 degrees)		4
	Extreme axis (−90–+180 degrees)		–
Low voltage (<0.5 mV in QRS amplitude)		15	
Chamber hypertrophy or enlargement		10	
	Left atrial dilatation/hypertrophy		–
	Right atrial dilatation/hypertrophy		–
	Left ventricular dilatation/hypertrophy		9
	Right ventricular dilatation/hypertrophy		1
Atypical ST‑T abnormalities		31	
	Non-specific ST‑T abnormalities		17
	Scooped ST‑T		2
	Flat T waves		10
	Tall T waves		2
Typical ST‑T abnormalities suggesting ischaemia or injury		2	
Acute or recent myocardial infarctions (MI)		1	
	Acute or recent anterior MI		1
(Suspected of) old myocardial infarctions		45	
	Old MI (anterior/inferior/posterior/lateral/not otherwise specified)		7
	Pathologic Qs		38
T‑wave inversion		31	
Pacemaker rhythm		2	
R‑wave abnormalities		23	
	Slow R progression		20
	Tall R wave		3
Prolonged QT interval		3	
Total		477	

General practitioners reported 477 ECG abnormalities in 144/852 ECGs; 111 cases concerned new abnormalities

*AV* atrioventricular, *ECG* electrocardiogram, *MI* myocardial infarction, *ST‑T* ST-segment and T‑wave

**Table 2:** Numbers (percentages) of abnormalities found and management actions taken by GPs after having performed an ECG

	ECGs performed for a specific indication during programmatic CVRM (*n* = 74)	Routine ECG at the start or in the course of programmatic CVRM (*n* = 778)	All ECGs (*n* = 852)
*New abnormalities found* *Total [95% CI]*	*18 (24.3%) [16.0–35.2%]*	*93 (12.0%) [9.9–14.4%]*	*111 (13.0%) [10.9–15.5%]*
*Management actions*			
Referral to cardiologist	5 (6.8%)	13 (1.7%)	18 (2.1%)
Additional diagnostics	3 (4.1%)	14 (1.8%)	17 (2.0%)
Second ECG	1 (1.4%)	11 (1.4%)	12 (1.4%)
Medication alteration	3 (4.1%)	8 (1.0%)	11 (1.3%)
Reassurance	1 (1.4%)	1 (0.1%)	2 (0.2%)
*Total [95% CI]*	*13 (17.6%) [10.0–28.5%]*	*47 (6.0%) [4.5–8.0%]*	* 60 (7.0%) [5.5–9.0%]*^*a*^

Compared to routine ECGs at the start or in the course of programmatic CVRM care, more new abnormalities (24.3% vs. 12.0%, *p* = 0.0093) and more management actions (17.6% vs. 6.0%, *p* = 0.0065) were reported in ECGs performed for a specific indication during programmatic CVRM care

*GP* general practitioner, *ECG* electrocardiogram, *CI* confidence interval, *CVRM* cardiovascular risk management

^a^ In 54/852 patients (6.3%), 60 management actions by GPs were registered

In 54/852 cases (6.3%), 60 new management actions were undertaken (most frequently referral to cardiologist (30.0%) and additional diagnostic testing (28.3%)). As compared with routinely performed ECGs at baseline or during follow-up, ECGs performed for a specific indication significantly more often evoked a new management action by the GP (17.6% vs. 6.0%, *p* = 0.0065, Tab. 2).

### The expert panel’s assessment

The expert panel assessed 300 ECGs: all 265 cases for which GPs reported abnormalities, complemented with a random sample of 35 ECGs evaluated as normal by the GPs. Most common clinically relevant ECG abnormalities described by the expert panel were previous myocardial infarction (55/300), relevant intraventricular conduction abnormalities (left and right bundle branch block (43/300) and atrial fibrillation (35/300).

**Fig. 3:**
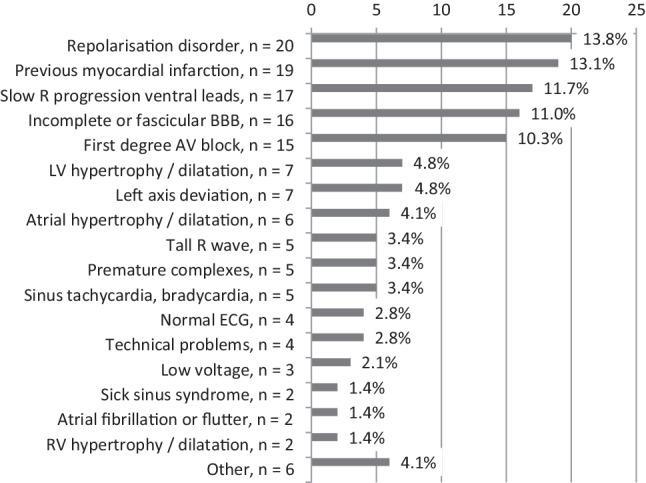
Number of ECG abnormalities missed or incorrectly interpreted by general practitioners and percentages of total number of 145 misinterpreted ECG abnormalities. In 97/297 ECGs assessable for the expert panel, the expert panel identified 145 misinterpretations. Other (*n* = 6) included: sinus node arrhythmia, extreme axis deviation, right bundle branch block, aberrant rhythm, ectopic atrial rhythm, 2nd degree AV block (each occurring once). *AV* atrioventricular, *BBB* bundle branch block, *ECG* electrocardiogram, *GP* general practitioner, *LV* left ventricular, *RV* right ventricular

A major disagreement on the interpretation of the ECG was present in three cases in which the expert panel assessed the ECG as normal whereas the GP had reported pathologic findings and nine cases vice versa (false negative rate 9/35, 26%). In three of these nine cases, the expert panel even proposed a management action. Overall, the expert panel considered 200/297 ECGs to be correctly interpreted by the GPs (67%), including 37/41 (90%) of ECGs considered ‘normal’ by the expert panel (Tab. 3). The GPs missed only one case of atrial fibrillation (35/36 cases of atrial fibrillation correctly recognised, 97%). Among 145 relevant misinterpretations by GPs, repolarisation disorder (20 cases), previous myocardial infarction (19 cases), slow R progression ventral leads (17 cases), incomplete or fascicular bundle branch block (16 cases) and first degree AV block (15 cases) were most common (Fig. 3).

**Table 3:** Agreement between general practitioner and expert panel concerning ECG interpretations and management actions for three ECG categories (normal, borderline, pathologic)

Panel’s diagnostic ECG category (3-point scale)			Agreement on ECG interpretation	Agreement on management actions
	Number (percentage) assessable for ECG interpretation	Number (percentage) assessable for management implications	Percentage [95% confidence interval]	Percentage [95% confidence interval]
– Normal	41 (13.8%)	41 (14.3%)	90 [81–100]	93 [84–101]
– Borderline	98 (33.0%)	96 (33.4%)^b^	67 [58–77]	76 [67–84]
– Pathologic	158 (53.2%)^a^	150 (52.3%) ^b^	61 [54–68]	66 [58–74]
Total	297 (100%) ^a^	287 (100%) ^b^	67 [61–72]	74 [68–79]

*ECG* electrocardiogram

^a^ 3 cases in the category ‘pathologic’ were excluded, because the panel determined the interpretation as ‘not assessable’

^b^ 13 cases were excluded, 2 in the category ‘borderline’ and 11 in the category ‘pathologic’, because the panel determined the management implications as ‘not assessable’

Regarding patient management, the overall agreement between expert panel and GPs was 74% and the agreement in the ‘normal’ ECG category was 93% (Tab. 3). In 76 cases, the expert panel’s preferred management actions differed from the GP’s management (additional diagnostic evaluation in 33 cases (echocardiography in 28 cases), different medication in 18 cases, different referral policy in 12 cases, other options including repeating the ECG because of bad quality and better hypertension control in 13 cases).

## Discussion

### Main findings

We evaluated the performance of GPs using ECG diagnostics in programmatic CVRM and diabetes care. Of 852 ECGs, 74 (9%) were recorded for a specific reason such as irregular pulse, chest pain, palpitations, adjustments or start of medication and treatment-resistant hypertension. New abnormalities were present in 111 cases (13.0%). In 54 cases (6.3%), the ECG led to a new management action by the GP. Whereas routinely recorded ECGs showed 12.0% abnormalities, and led to management actions in 6.0%, ECGs recorded for a specific reason yielded 24.3% abnormal ECGs and in 17.6% management actions followed. As compared to the EP, the GPs scored moderately well on quality of ECG interpretation (67% agreement) and patient management (74% agreement). GPs performed best in the interpretation of ECGs that were normal according to the expert panel and ECGs showing atrial fibrillation.

### Performance of GPs

Overall, the GPs’ performance was moderate compared with the expert panel: in our earlier study on daytime ECGs in symptomatic patients, agreement on interpretation (83.8%) and management (88.3%) was higher than in the current study (67% and 74% respectively) [24].

In the current study, the GPs accurately detected atrial fibrillation (97% agreement) and ECGs deemed ‘normal’ by the expert panel (90% agreement). Similar values have been described in other studies, ranging from 81 to 94% [6, 26]. ‘Borderline’ ECGs were properly interpreted in 67% and ‘pathologic’ ECGs in 61% of the cases. In literature, the agreement on key findings varies from 59 to 70% [27, 28]. In the current study, GPs had most difficulties recognising R‑wave abnormalities, atrial or ventricular enlargement, previous MI, and atypical ST‑T abnormalities. Although the sample size of ECGs judged normal by the GPs (*n* = 35) was low, the high false negative rate (26%) justifies emphasising the possibility of finding abnormalities in routinely recorded ECGs to GPs.

Disagreement of the expert panel concerning patient management mostly included referral policy, prescription of medication and additional diagnostic measures, mainly echocardiography. Literature shows similar findings: the majority of symptomatic patients are appropriately referred to the hospital [28].

### ECG in CVRM and diabetes care: is it useful?

Twenty GPs performed an average of 42 ECGs per GP in one year in the context of programmatic CVRM and diabetes care. This illustrates the high volume of ECGs in this setting. Only a minority of these ECGs (9%) was recorded for a specific reason. In these ECGs, GPs found more abnormalities and initiated more management actions as compared to routine ECGs.

The number needed to screen (NNS) for any abnormality was four in case of a specific indication and eight in routine ECGs. The NNS for a management consequence was six for specific ECGs and 17 for routine ECGs. Although the yield of screening for atrial fibrillation was not a main objective of this study, we observed 12/852 cases (1.4%) of newly found atrial fibrillation. Six atrial fibrillation cases were found in 74 ECGs recorded for specific indications (‘opportunistic screening’, 8.1% yield, NNS 12.3) and six cases were observed in 778 routine ECGs (‘population screening’, yield 0.77%, NNS 130). This NNS of 130 seems promising, since a recent meta-analysis found an NNS of 170 to be possibly cost-effective and efficiency is even higher if ECGs are recorded only after abnormal findings upon pulse palpation or usage of modified blood pressure monitors [29].

These examples illustrate that making ECGs in the context of CVRM and diabetes care *only for specific indications *seems more efficient due to a lower absolute volume of ECGs with a higher percentage of abnormalities detected. However, a number of relevant abnormalities (e.g. atrial fibrillation) will then be missed. Previously, even routine ECGs in CVRM were estimated to be of added value to prevent death (estimated NNS = 260 to prevent one cardiovascular death in ten years) [5]. Yet, further studies are necessary to assess the factual benefit of electrocardiography in CVRM.

### Strengths and limitations

We studied an unselected and rather large sample of 852 real life ECGs in the context of programmatic CVRM and diabetes care. At the time the GPs requested and interpreted the ECGs, they were unaware of the current study. Therefore, we regard our findings representative for the ECG performance of selected GPs who make ECGs and feel competent enough to participate in a study such as ours. Our panel assessment was based on at least two panel members’ independent judgements. The expert panel disagreed with the management action only if there were clear directives for this decision. There are no reasons suggesting that the pattern of recording ECGs in CVRM in primary care has changed between the study year (2016) and the present.

On the other hand, because of the retrospective design of the study, documentation by GPs occasionally was incomplete. Moreover, the expert panel judged the cases only on paper, thereby possibly missing clinical or contextual factors that may have influenced the GP’s decision. Both weaknesses may have influenced the panel evaluation, possibly leading to lower agreement figures. Our study had not enough statistical power to compare ECGs in CVRM versus diabetes care.

### Conclusions

Overall, ECGs recorded by GPs in the context of programmatic CVRM and diabetes care yield new abnormalities in 12% of the cases, and lead to new management actions by GPs in 6%. Both outcomes are higher for ECGs recorded for a specific reason (24% and 18% respectively). Educating GPs seems necessary, since they perform less well in interpreting and managing ECGs made in the context of CVRM and diabetes care than in ECGs performed in symptomatic patients.

## Caption Electronic Supplementary Material

**Supplementary Table 1** Diagnostic ACC/AHA ECG categories [25]

**Supplementary Table 2** Characteristics of the 20 participating general practitioners

**Supplementary Table 3** Population characteristics of patients in programmatic cardiovascular risk management or diabetes care in whom GPs had performed an ECG
