# Supplementary material for: Interpretations of and management actions following ECGs in programmatic cardiovascular care in primary care: A retrospective dossier study
Source: Neth Heart J. 2020 Feb 19;28(4):192–201. doi: 10.1007/s12471-020-01376-3 (PMC7113334; doi:10.1007/s12471-020-01376-3)
Supplement: Supplementary file 1 — Supplementary Table 1 Diagnostic ACC/AHA ECG categories [25] [file 12471_2020_1376_MOESM1_ESM.docx]

**Supplementary Table 1** Diagnostic ACC/AHA ECG categories (26)

| Normal | Borderline | Pathologic |
| --- | --- | --- |
| normal ECG  no abnormalities  technical problems (leads misplaced, artefacts)  sinus node arrhythmias caused by breathing  SVES or PACs (1 SVES/10 seconds)  VES (1 VES / 10 seconds)  horizontal axis  vertical axis  physiological Q waves | sinus tachycardia (>100/min)  sinus bradycardia (50-60/min)  sinus node arrhythmias (mild)  ectopic atrial rhythm  SVES or PACs (≥2 SVES/10 seconds)  VES (2 VES/10 seconds)  1st degree AV block  iRBBB  LAFB  LPFB  LVH  subtle ST-T abnormalities  atypical ST-T abnormalities  juvenile ST-T abnormalities  isolated Q in III  low voltage (<0,5mV in QRS amplitude)  slow R progression  PTa depression (pericarditis) | sinus bradycardia (<50/min)  sinus arrhythmias (>50% variation in atrial function)  atrial fibrillation  atrial flutter  fibrilloflutter  VES (≥3 VES / 10 seconds)  ventricular fibrillation  2nd and 3rd degree AV block  LBBB  RBBB  RVH  left axis (+90° - +180°)  right axis (-30° - -90°)  typical ST-T abnormalities suggesting ischaemia or injury (ST depressions, ST elevations, stretched ST-T, T inversion)  signs of old infarctions (pathologic Q waves, loss in R-wave progression)  pacemaker |
|  | | |

This list guided the panel members in choosing a diagnostic ECG category on a three-point scale. However, they could make their own decision, e.g., the co-occurrence of multiple borderline abnormalities was diagnosed as ‘pathologic’ by the panel.

*ACC/AHA* American College of Cardiology/American Heart Association, *AV* atrioventricular, *ECG* electrocardiogram, *(i)RBBB* (incomplete) right bundle branch block, *LAFB* left anterior fascicular block, *LBBB* left bundle branch block, *LPFB* left posterior fascicular block, *LVH* left ventricular hypertrophy, *PACs* premature atrial complexes, *RVH* right ventricular hypertrophy, *ST-T* ST-segment and T‑wave, *SVES* supraventricular extra systole, *VES* ventricular extra systole
