# Supplementary material for: Interpretations of and management actions following ECGs in programmatic cardiovascular care in primary care: A retrospective dossier study
Source: Neth Heart J. 2020 Feb 19;28(4):192–201. doi: 10.1007/s12471-020-01376-3 (PMC7113334; doi:10.1007/s12471-020-01376-3)
Supplement: Supplementary file 2 — Supplementary Table 2 Characteristics of the 20 participating general practitioners [file 12471_2020_1376_MOESM2_ESM.docx]

**Supplementary Table 2** Characteristics of the 20 participating general practitioners

| Variable |  | Total  (*n*=20) |
| --- | --- | --- |
| General | Number of males (%) | 10 (50%) |
|  | Mean age in years [range] | 47 [28–61] |
| Expertise | Mean years of service [range] | 17 [1-33] |
|  | Number of GPs who had participated in an ECG training program (%) | 18 (90%) |
|  | Number of GPs who had been cardiology residents* (%) | 8 (40%) |
| ECG volume | Mean number of self-reported ECG interpretations per month [range] | 14 [1-80] |
| ECG use in programmatic CVRM or DM care | Number of GPs who routinely make baseline ECGs in programmatic CVRM and DM care (%) | 11 (55%) |
|  | Number of GPs who routinely make follow-up ECGs in programmatic CVRM and DM care (%) | 6 (30%) |
|  | Number of GPs who make ECGs for a specific reason during programmatic CVRM and DM care (%) | 20 (100%) |

* Cardiology residents included residency at cardiology (*n*=7) and cardiothoracic surgery (*n*=1).

*GPs* general practitioners, *ECG* electrocardiogram, *CVRM* cardiovascular risk management, *DM* diabetes mellitus
