# Supplementary material for: Interpretations of and management actions following ECGs in programmatic cardiovascular care in primary care: A retrospective dossier study
Source: Neth Heart J. 2020 Feb 19;28(4):192–201. doi: 10.1007/s12471-020-01376-3 (PMC7113334; doi:10.1007/s12471-020-01376-3)
Supplement: Supplementary file 3 — Supplementary Table 3 Population characteristics of patients in programmatic cardiovascular risk management or diabetes care in whom GPs had performed an ECG [file 12471_2020_1376_MOESM3_ESM.docx]

Supplementary Table 3 Population characteristics of patients in programmatic cardiovascular risk management or diabetes care in whom GPs had performed an ECG

| Variables | | All ECG cases (*n*=852) | | | ECG cases assessed by the expert panel (*n*=300)* | | |
| --- | --- | --- | --- | --- | --- | --- | --- |
|  |  | Total | Male | Female | Total | Male | Female |
| General | Number of cases (row %) | 852 (100%) | 463 (54.3%) | 389 (45.7%) | 300 (100%) | 191 (63.7%) | 109 (36.3%) |
|  | Mean age in years  (± SEM [range]) | 66.37  ± 0.377 [25-90] | 65.66  ± 0.525 [25-90] | 67.23  ± 0.539 [29-90] | 69.11  ± 0.639 [25-90] | 68.5  ± 0.824 [25-90] | 70.18  ± 1.002 [29-90] |
| CVD events / risks | Mean number of known CVD events in history per case  (± SEM [range]) | 0.33  ± 0.023 [0-4] | 0.35  ± 0.032 [0-3] | 0.30  ± 0.034 [0-4] | 0.49  ± 0.045 [0 - 4] | 0.48  ± 0.055 [0-3] | 0.52  ± 0.079 [0-4] |
|  | Number of cases with 1 or more CVD events in history  (row % / column %) | 191  (100% / 22.4%) | 114  (59.7% / 13.4%) | 77  (40.3% / 9.0%) | 103  (100% / 34.3%) | 64  (62.1% / 33.5%) | 39  (37.9% / 35.8%) |
|  | Mean number of known CVD risk factors per case  (± SEM [range]) | 2.38  ± 0.038 [0-6] | 2.41  ± 0.053 [0-6] | 2.33  ± 0.055 [0-6] | 2.39  ± 0.064 [0-6] | 2.38  ± 0.081 [0-6] | 2.40  ± 0.104 [0-5] |
|  | Number of cases with DM as risk factor (row % / column %) | 224  (100% / 26.3%) | 137  (61.2% / 16.1%) | 87  (38.8% / 10.2%) | 90  (100% / 30%) | 56  (62.2% / 29.3%) | 34  (37.8% / 31.2%) |
| Reason consultation | CVRM - number  (row % / column %) | 655  (100% / 76.9%) | 346  (52.8% / 40.6%) | 309  (47.2% / 36.3%) | 222  (100% / 74%) | 143  (64.4% / 74.9%) | 79  (35.6% / 72.5%) |
|  | DM management - number  (row % / column %) | 197  (100% / 23.1%) | 117  (59.4% / 13.7%) | 80  (40.6% / 9.4%) | 78  (100% / 26.0% | 48  (61.5% / 25.1%) | 30  (38.5% / 27.5%) |
| Indication for ECG | Routine baseline - number  (row % / column %) | 49  (100% / 5.8%) | 28  (57.1 / 3.3%) | 21  (42.9% / 2.5%) | 13  (100% / 4.3%) | 9  (69.2% / 4.7%) | 4  (30.8% / 3.7%) |
|  | Routine follow-up - number  (row % / column %) | 729  (100% / 85.6%) | 399  (54.7% / 46.8%) | 330  (45.3% / 38.7%) | 254  (100% / 84.7%) | 161  (63.4% / 84.3%) | 93  (36.6% / 85.3%) |
|  | Specific indication - number  (row % / column %) | 74  (100% / 8.7%) | 36  (48.6% / 4.2%) | 38  (51.4% / 4.5%) | 33  (100% / 11.0%) | 21  (63.6% / 11.0%) | 12  (36.4% / 11.0%) |

* All 265 abnormal ECGs plus a random sample of 35/587 normal ECGs.

*GPs* general practitioners, *ECG* electrocardiogram, *CVD* cardiovascular disease, *CVRM* cardiovascular risk management, *DM* diabetes mellitus, *SEM* standard error of the mean
